# The Psychological Structure of Loneliness

**DOI:** 10.3390/ijerph19031061

**Published:** 2022-01-18

**Authors:** Axel Seemann

**Affiliations:** Department of Philosophy, Bentley University, 175 Forest Street, Waltham, MA 02452, USA; aseemann@bentley.edu

**Keywords:** loneliness, experience, emotion, perception, cognition, intersubjectivity

## Abstract

Despite the current surge of interest in loneliness, its health consequences, and possible remedies, the concept itself remains poorly understood. This paper seeks to contribute to a more fully worked out account of what loneliness consists in. It does this by stressing that loneliness always has an experiential component and by introducing a simple psychological structure to analyze the experience. On this basis, it suggests that we can distinguish between three ways of thinking about the phenomenal dimension of loneliness. There are objectivist views that seek to understand loneliness by a description of its intentional object, subjectivist views that consider its holistic relation to other aspects of the sufferer’s psyche, and embodied and enacted views that focus on the relation between the lonely person’s mental life and her social environment. The aim is not to adjudicate between these views or to suggest that they are mutually exclusive. Rather, this paper recommends a pluralistic framework on which all three approaches have something to contribute to a fuller understanding of the condition and may be of use in devising measures aimed at improving sufferers’ health.

## 1. Introduction

Loneliness is a pervasive public health concern, significantly correlated with increases in health problems and mortality rates [1,2]. A rapidly growing literature is aimed at identifying methods to diagnose the condition and provide help to sufferers (see [3] for an overview). For this to be possible, a plausible description or definition of loneliness has to be in place. Several such definitions are available; see [4] for an overview of definitions and [5] for a typology of interventions for loneliness. They are accompanied by corresponding measurement systems such as the UCLA Loneliness Scale [6]. In contemporary psychological research, many descriptions frame loneliness as a perceived discrepancy between desired and available social connections (e.g., [2,7]; p. 839). This seems uncontroversial as a first step, but questions arise immediately: what is meant by a “social connection” (or “social interaction” or “social relationship”)? How do you think about its absence? How do you make precise the notion of an “unpleasant experience” or a “distressing feeling”?

These questions are of philosophical import, but their relevance does not stop there. How they are approached has consequences for the design of measures designed to help sufferers. Different ways of thinking about loneliness bring with them different recommendations for therapy or changes in the affected person’s social environment. However, and in contrast to the flourishing debate in psychology about the diagnosis and remedy of loneliness, the discussion of its conceptual dimension is in its infancy. Historically, philosophers have thought about loneliness as an existential (e.g., [8]) or political concept [9]. It is only very recently that they have framed the condition as a mental health concern ([10,11]. It is hence telling that Motta’s [4] overview of theoretical approaches to loneliness builds on a summary of psychological concepts published several decades ago [12]. The research available on loneliness in contemporary philosophy is not yet robust enough to allow for a qualified discussion of its conceptual dimension.

The present paper is intended to contribute to this discussion by raising some questions that a full-fledged philosophical theory of loneliness, understood as a condition that is detrimental to the sufferer’s health, would have to address. After a brief classification of issues that arise from a widely used definition of loneliness in psychology, this paper draws on some long-standing debates in the philosophy of mind to show how these debates can inform the experiential dimension of loneliness research. Its aim is not to develop a comprehensive theory of loneliness or even its phenomenal aspect. It does not offer a framework that could be classified along the lines of Perlman and Peplau’s [12] conceptual or Mann, Bone, and Lloyd-Evans’s [5] practical typologies of loneliness. It merely helps prepare the ground for the development of a theory that incorporates the phenomenal dimension of loneliness and, in doing so, highlights the importance of the sufferer’s experience for the design of diagnostic and remedial work.

## 2. Three Dimensions of Loneliness

Here are two relatively recent definitions of loneliness in widely cited psychological research papers:

1. Loneliness is “the unpleasant experience that occurs when there is a subjective discrepancy between desired and perceived availability and quality of social interactions” ([7] p. 839).
2. Loneliness is “a distressing feeling that accompanies the perception that one’s social needs are not being met by the quantity or especially the quality of one’s social relationships” ([2]).

Variants of these two definitions are popular in the academic and popular literature on loneliness (consider, e.g., the definition of loneliness in the Encyclopedia Britannica as the “distressing experience that occurs when a person’s social relationships are perceived by that person to be less in quantity, and especially in quality, than desired” [13]). In particular, they are commonly adopted by writers who see loneliness as a condition that is detrimental to the sufferer’s mental and physical health and that thus benefits from remedial intervention. The two definitions, though not quite identical, represent what I shall call the “standard view” of loneliness. They have at least three features in common. They both understand loneliness as an experience—that is, a mental state, event, or relation that can be described in terms of its subjective characteristics. They both agree, secondly, that the experience in question has a negative valence: it is “unpleasant” or “distressing”. Thirdly, they agree that the experience is about something: it presents, or represents, a discrepancy between the social relationships that would meet one’s social needs and those that one does in fact enjoy, or that are available. These three features are associated with distinct philosophical areas of investigation. They can be classified along the following lines:

### 2.1. The Phenomenal Dimension

This dimension of loneliness research covers all questions that have to do with the first-person, subjective aspect of the phenomenon. Along the lines of the standard view, loneliness always has an experiential aspect to it. But more will need to be said for a well-worked out theory: what kind of experience is loneliness? Is it appropriately described as a feeling or an emotion (see Damasio [14] for a prominent account of the distinction)? If so, which (if any) of the extant theories of emotion in the philosophical literature is well placed to capture the experience of loneliness (see Scarantino and de Sousa [15] for an overview of theories of emotion)? Relatedly, should we think of the experience as having success conditions, and that (correspondingly) a token experience can misrepresent its intentional object? Or should we deny that one can feel lonely when in fact one is not? How these questions are answered will have considerable impact on the design of remedial measures: for instance, if the sense of being lonely has correctness conditions, then therapeutic work may promisingly highlight the falsidical character of a sufferer’s experience. If, however, feeling lonely is taken to be sufficient for loneliness, such an approach would be grossly misguided.

### 2.2. The Social Dimension

A separate set of question arises with regard to the “social relationships” or “social interactions” that a person is experiencing as lacking in loneliness. There are many kinds of social interactions and social relationships, and not all of them are equally relevant in the alleviation of the condition. More needs to be said about what is meant by these notions. Who are the partners in the relevant kinds of social interactions? Do they have to be exercised in person, or can virtual reality help (e.g., [16])? Is there an important bodily aspect to them? How does the developmental role of social interaction in the regulation of a person’s emotional life bear on a theory of loneliness? What factors other than interaction are important to substantiate the kind of social relationship whose enjoyment helps alleviate the condition? Depending on how you answer these questions, diagnostic and remedial measures will again vary considerably, and hence a more focused discussion of the social dimension of our understanding of loneliness is indispensable.

### 2.3. The Normative/Cognitive Dimension

On the standard view, the experience of loneliness arises because of a perceived discrepancy between the relationships (however conceived) needed for a satisfactory social life and those that are currently available to the sufferer. So there is a normative aspect to the experience: it reflects a lack or an absence of something that should be there but is not (I am using “normative” here in the philosophical rather than the psychological sense; thanks go to a reviewer for highlighting the distinction). Then at least two questions arise. The first is how to think of what it is that is perceived to be missing—what the intentional object of loneliness is. Is it meaningful relationships themselves, or is it an abstract object such as a friendship or another “social good” that is instantiated by but not the same as the right kinds of social relationships [11]? The second question, which bridges the normative and phenomenal dimensions, is how the absence of the relationships that would alleviate loneliness is present to the sufferer. What does it mean to experience a loss, or an absence? Does the experience of loneliness operate against a background, or a memory, of how one’s social relationships should be, or perhaps once were? Is this normative background or memory present in the sufferer’s experience, or does it register cognitively? Once again, answers to these questions are not merely of philosophical interest. They are directly relevant for remedial work: if the theorist can explain how the norm against which an experience of loneliness arises is present in experience or cognition, the practically oriented researcher will be much better placed to work out what can be done to minimize the perceived discrepancy between a sufferer’s actual and desired social relationships.

## 3. Experience as a Necessary Condition of Loneliness

The above taxonomy of questions arising for a fully developed philosophical theory of loneliness helps situate the present paper in the larger context of loneliness research. The project squarely falls into the first category: it is concerned with the phenomenal dimension of loneliness. Since it restricts itself to highlighting the relevance of some discussions in the philosophy of mind for such an investigation, it remains largely silent on the question of what loneliness is. Rather, it elaborates on three distinct ways of thinking about its phenomenal aspect. These ways pick up on debates in the philosophy of perception. The aim is to show that we can draw on these debates to better understand loneliness in practically useful ways.

Psychologists distinguish between loneliness as an “objective” and a “subjective” condition (e.g., [7]). Some people are objectively socially isolated: they have little social contact with others. Some people are subjectively lonely: they report feeling alone, in the sense of not having as much social contact as they would want. As is now well-known, the two kinds are not reliably correlated [17]. Not every hermit is lonely but some socialites are. Distinguishing between objective and subjective forms of loneliness thus tracks an intuitively obvious point. But it is important to think carefully about the exact difference that is being tracked. Begin with the consideration that on the standard view, thinking about loneliness always requires thinking about a person’s subjective life—their experience, the felt quality of their existence (this consideration is explicitly acknowledged in Hawkley and Cacioppo [2]). If so, it is not promising to distinguish between a purely objective kind of loneliness that is measurable in terms of the number and quality of a person’s social contacts and a subjective kind that tracks the sufferer’s experience. A better way to draw the distinction is to take it that the subjective dimension establishes a necessary condition of loneliness: a person can be lonely only if she feels lonely. This necessary condition does not require that the sufferer be cognitively aware of her loneliness: her experience need not gives rise to the knowledge that she is lonely. But it does rule out the possibility that the sufferer is (objectively) lonely without experiencing herself as lonely. On this picture, a person cannot be lonely without this fact being reflected in her mental life. By contrast, the absence of social connection is neither a necessary nor a sufficient condition of loneliness: loneliness begins and ends with experience.

This may seem an uncontroversial point: obviously loneliness has an experiential aspect to it. Otherwise, the important distinction between loneliness and the adjacent condition of solitude, in which a person also has few social connections but experiences this fact as beneficial, collapses (historically, the adjacent concepts of loneliness and solitude were not as clearly demarcated as they were today, as a reviewer pointed out; see Vincent [17] and Alberti [18] for recent overviews). But framing the relation between the subjective and objective aspects of loneliness in terms of a necessary condition highlights that even where objective social isolation can be shown to negatively affect a person’s physical health, there has to be an intermediate psychological component for the person to qualify as lonely: loneliness, on any account, is not a straightforward physiological condition whose causes can be directly traced to environmental factors without detour via the mental domain (see Motta [4] p. 74, for the related point that it would be a mistake to equate loneliness with social isolation). Consider this formal rendering of the necessary condition that a person has to meet if she is to be capable of loneliness:

(NEC) A person can suffer from loneliness only if she has a subjective life, and if illuminating her condition requires reference to her subjective life.

(NEC) is very broad. It does not take a view on how we should think about the notion of a “subjective life”, and it does not take a view on the shape or role of experience in possible accounts of loneliness. It is not presented as a novel or original insight for loneliness research; in fact, it amounts to little more than a platitude. But it brings out the point that even where objective social isolation is identified as the cause of, or reason for, someone’s loneliness, a full investigation of the condition requires reference to the sufferer’s experience.

Two discussions in the philosophy of mind are useful here. The first is the long-standing debate about the qualitative aspect of experience—its subjective dimension or “what’s-it-like”-ness—that is largely but not exclusively conducted in phenomenology. Tietjen and Furtak [19] investigate this subjective aspect of loneliness. The second is the debate about the internal structure of a person’s mental life and its relation to the environment in which she operates. To my knowledge there is, as yet, no work that relates this debate to loneliness research. Yet this is an important area of investigation for a philosophical theory of loneliness, as I hope to show in what follows. Begin by considering ordinary visual experience: suppose a perceiver has a visual experience of an apple that is placed on the table before her. One way of conceptualizing the experience is to say that it is directed at or about the apple and that it can succeed or fail in correctly presenting or representing the apple to the perceiver. Alternatively, one can think that the connection between experience and its object is less direct than this way of putting things allows: it is not only that experience is “in the head”; the direct object of the experience are sensory data that are in the head also. Or one can take it, thirdly, that when a subject’s visual experience succeeds in presenting the apple as it is to her, she is standing in a direct phenomenal relation to the object that is cognitively and (on some views) phenomenally distinct from the experience she would be suffering if she were, say, undergoing a hallucination. 

These three ways of conceptualizing the relation between mind and world motivate three distinct approaches for the quest to understand the experiential dimension of loneliness (this is really all that the parallel with visual experience is meant to accomplish here; there is no suggestion that the three approaches to thinking about the phenomenal aspect of loneliness are wedded to particular theories of perception). One can take it that loneliness is or involves an experience that is directed at or about something, and that making sense of the experience requires you to consider the intentional object that it is directed at and the way (the “attitude”) in which this directedness manifests itself. I call such views “objectivist” or “intentionalist”. Secondly, one could seek to understand the experience of loneliness as what you might call a “mood”—a fundamental coloring of a sufferer’s subjective life that requires the theorist to reflect on facts that are internal to her psychology. Though conceiving of loneliness as a mood does not rule out the possibility that there are external factors that influence and help explain the sufferer’s mental life (such a view would not be at all attractive), coming to understand a mood requires that one think primarily about the larger psychological context in which it occurs and that it affects. I call such views “subjectivist”. A third way of thinking about loneliness is in terms of an embodied and enacted relation between the sufferer and her environment. On such a view, a creature’s relation to its environment is constituted in action upon and interaction with it, and its interactions shape both the way its objects are presented to the sufferer and how he comes to understand his own place in it. I call such views, “relationist” or “embodied”. 

In the philosophy of perception, these three views are competitors: either the objects of experience are mind-independent, or they are not; either the body plays a constitutive role in the shaping of experience, or it does not. I am not suggesting that the theorist about loneliness is faced with a similarly binary choice. For instance, one can plausibly suppose that some kinds of loneliness are, or involve, object-directed mental states while others are general moods. Consider the difference between someone who feels lonely at a party full of strangers; someone who feels lonely because his partner has died; and someone whose chronic sense of loneliness pervades all areas of her life. The first two examples are, in different ways, object-involving, the third one qualifies as a mood. But each person qualifies as lonely in virtue of conforming to the standard view of loneliness outlined in Section 2. Pluralism seems an advisable starting point for a reflection on how to conceive of this complex condition. Hence, the following is not meant as a sketch of rival conceptions of loneliness but rather of different approaches that each may be useful in particular cases and contexts.

## 4. The Psychological Structure of Loneliness

### 4.1. Intentionality and Objectivism

Intentionalism in the philosophy of perception is the view that perceptual experience has an “intentional object” and that the experience is directed at or about that object (e.g., [20,21]). The intentionalist view of the mind in contemporary philosophy goes back to Brentano [3]; for a recent account of the concept in phenomenology see Krueger [22]; for an account of the notion as it pertains to the emotions see Ratcliffe [23]. The view can seem almost trivially true: when one has an experience of an apple on the table, the experience is about, or directed at, that apple (see Searle [24] for a classic account of intentionality in the philosophy of perception). It can misrepresent the apple: perhaps what one is seeing looks like an apple but is really a pear, or perhaps one is hallucinating an apple in the absence of any visual object. The resulting picture draws a stark distinction between a perceiver’s mental state and the object it is directed at. It can be put to use for a conception of loneliness. One way to substantiate the approach is to take it that the intentional object of an emotion can be specified physiologically. William James [25] suggested that emotions are reports on physiological change. Building on this view, Prinz [26] argues that emotions are perceptions of bodily change that have specific functions and valence markers. Thus, fear is the perception of a racing heart, has the function of being elicited by danger and signals “less of this!”, which motivates avoidant action. A related approach is being advocated by Cacioppo’s and Patrick’s prominent view that loneliness is “social pain” [27]. Loneliness is conceptualized as a felt response to the brain processes that are triggered by social isolation, whose unpleasant character motivates the sufferer to alleviate the condition.

A very different way to give substance to the intentionalist approach is Roberts and Krueger’s [11] proposal to understand loneliness as the experience of an absent social good. Then one can make sense of the condition by thinking about the notion of a social good and the attitude of the sufferer towards it (and the inquiry then crosses over from the phenomenal into the social dimension of loneliness research identified in Section 2). In what follows, I shall work with Roberts’s and Krueger’s account to draw out some implications of an intentionalist approach to loneliness. But the lessons are meant to be general: they are intended to apply to various possible ways of thinking about loneliness in intentionalist terms. One distinctive feature of Roberts’s and Krueger’s account is that it treats loneliness as the experience of an absence. This is an intuitively plausible suggestion: when you are lonely, something is missing. Roberts and Krueger make two moves to account for the experience of loneliness. They posit that there is a range of social goods that the sufferer desires but that she realizes to be out of reach, such as “companionship, moral support, physical contact and affection, sympathy, trust, romance, friendship, and the opportunity to act and interact” ([11] p. 7). These social goods constitute the object of the lonely person’s emotion. Secondly, the person has a “pro-attitude” towards these goods ([11] p. 10)—she actually desires (some of) them. At the same time, she realizes that they are out of reach, and this realization gives rise to the painful experience of loneliness. One question that arises for the account (and, in similar form, for other intentionalist proposals) is how we should think of the good that is absent. Compare again a perceptual scenario: a perceiver enters her living room in which she expects to find an armchair by the bookshelf. But, startlingly, the armchair is missing: there is only an empty bit of rug where the armchair should be. For the perceiver to be surprised by the armchair’s absence, she has to be operating with an expectation that it be on the rug, in its habitual place. She has to be operating with a norm (the habitual outlay of the living room), and it is by comparison with this norm that her surprise arises (the idea that visual perception is normative is classically defended by Merleau-Ponty [28]).

An emotion that is brought about by the absence of its intentional object also has a normative element to it: the experience is explained by the unattainability of a desired state-of-affairs, such as the presence of companionship, friendship, or some other social good (this consideration belongs to the third dimension of loneliness research outlined in Section 2). But note the difference between the perceptual case and the conception of the object of loneliness as a social good. In the perceptual case, an actual state of affairs, instantiated by particular objects, constitutes the norm. In the case of loneliness, on Roberts’ and Krueger’s view, the norm is constituted by a formal object. This provokes the question of how the person comes to desire this good—few people feel lonely because of a perceived lack of some abstract conception of “friendship”, for instance. Perhaps someone is lonely because of a particular friendship she enjoyed and lost, or perhaps of a story about friendship that she has read about and that has made her realize what is missing in her life. This can make it seem as if the insistence on a social good as the object of an experience of loneliness were unduly cumbersome—would it not be simpler to say that the object of someone’s loneliness, in many cases at least, is an actual person, or group of persons, the loss of interactions with whom is painfully felt? But such an alternative account invites questions too: it cannot simply be the person, qua person, whose absence gives rise to loneliness. It has to be the interactions with her and what these interactions mean to the sufferer. Now an account is needed of these interactions and their meanings. And then it is beginning to look as if this meaning might well be captured by the notion of a social good after all. 

The upshot of this brief discussion is as follows: if one thinks of loneliness as being object-directed, one needs to say more about what this object consists in. This is not a trivial task; neither appealing to abstract objects such as friendship nor to particular persons is without its problems. The question of the conception of the object of someone’s loneliness has practical implications: what measures are undertaken to relieve the condition will depend on what the sufferer’s painful experience is about. Loneliness that is brought about by the loss of a particular person may require a different kind of help than loneliness that is about a general desire for companionship.

The second important dimension of this intentionalist account is that the subject exhibits a “pro-attitude” towards the object of her experience that is being frustrated (for a defense of the view that emotions are attitudes, see Deonna and Terroni [29]; for a recent discussion, see Rossi and Tappolet [30]). There is a question what this suggestion implies for the sufferer’s actions. The view that emotional attitudes are action-guiding is defended, e.g., by Deonna and Terroni [29] and in a different way by Goldie [31], who introduces the notion of “feeling towards”, a world-involving emotion that presents the environment in action-guiding ways (you may experience an icy stretch of road as slippery and thus step onto it with caution). What, though, should one say about the lonely person’s aptness to actively alleviate her condition? Cacioppo, Cacioppo, and Boomsma [32] suggest that loneliness evolved as a mechanism to enhance social connection, so the lonely person should be motivated to seek company (it should be noted that Cacioppo et al. do not claim that loneliness invariably motivates the sufferer to pursue social connections). On the other hand, you could think of deep loneliness as an experience that results in the sufferer leading an ever more solitary life. Thus, Roberts and Krueger [11] p. 16 suggest that chronic loneliness manifests itself in a lack of concern for the relevant social goods and that this results in an affective flattening in which people and environments cease to be presented as significant. More work is needed on the role of the attitude the lonely person displays towards the intentional object of her experience.

A brief closing consideration is to do with a much-discussed hallmark of intentionalist accounts of perceptual experience. The intentionalist is committed to the view that a perceiver can be in the same kind of mental state regardless of whether her experience of an external object is veridical. This consideration provokes the question of whether there is such a thing as an experience of apparent loneliness that misrepresents its object: the person experiences herself as lonely even though she is not, in fact, suffering from a lack of meaningful social connection, or social goods. Such a line of thought could develop in different directions. One option is to think that the lonely person may be mistaken about the object of her experience: perhaps it is not the lack of companionship but of love that makes her feel lonely. But you could also, more drastically, surmise that there are experiences of loneliness that the person ought not to have: someone might feel lonely without actually being lonely, and in that sense the experience is misguided; the person is not entitled to the experience. It is no doubt a delicate question whether that view has any substance.

### 4.2. Subjectivism and Moods

A different way of thinking about loneliness becomes available if you do not begin with the idea that loneliness has an object. Such an approach does not necessarily (though it may) amount to the claim that the experience of loneliness altogether lacks an object; more moderately, the suggestion may be that the character of loneliness is to be found in the sufferer’s general attitude towards her surroundings. Something like this view is suggested by our adverbial everyday use of the word “lonely”. When we say that someone feels lonely, we are suggesting a mode of experience that is not obviously like, for instance, being afraid. Typically at least, the fearful person is afraid of some particular thing. It is not obvious that the lonely person’s experience is about something in an even roughly corresponding way. Rather, you might think, the absence of social companionship has a pervasive effect on her mood: much or all of her mental life is colored by it. This is true particularly for chronic loneliness, in which the person may feel lost in the world quite independently of what she is doing and in which surroundings she is moving. 

Suppose loneliness is a mood, a fundamental coloring of experience. One can think of moods that are plausibly described as not having objects: a sense of bottomless fatigue or of nameless dread are examples. But it is not easy to see loneliness as that kind of mood. However one understands the notion, it is hard to deny that the very meaning of the term is in some way tied to the absence of others and is in that sense about something; take this aboutness away and it is not clear what we mean when we ascribe loneliness to someone. It may be more promising to think of loneliness as a mood without thinking of it as objectless. Goldie [31] suggests that moods are directed at the whole world. Applied to loneliness, the view might then be that the lonely person’s experience of her whole environment is pervaded by her sense of being alone wherever she goes. One can make particularly good sense of this proposal for instances of chronic loneliness, in which the person really may feel isolated not only from other people but everything else as well—institutions, countries, landscapes (such a view would not be far removed from the already mentioned way in which Krueger and Roberts understand chronic loneliness: a flattening of affect in which all social goods have lost their interest). There is a fine line here between this characterization and chronic depression, which also may feature an all-pervasive sense of isolation. Generally, there is an important and difficult question of how to think of loneliness in relation to depression: is it a kind of depression, or a different but related kind of psychological condition? The analysis of loneliness as a mood that is directed at the whole world makes the differentiation quite difficult, since one can describe deep depression as directed at the whole world also, and since its phenomenology—a sense of isolation, of lack of connection—can be described in similar terms too (for instance, Ratcliffe [33]) argues that depression is interpersonally structured).

A different way of understanding loneliness as a mood is available by appeal to Ratcliffe’s [34,35] notion of an “existential feeling”. Ratcliffe thinks of such feelings as all-encompassing moods, outlooks that constitute “a sense of how one finds oneself in the world as a whole” that shapes one’s sense of possibility. Different existential feelings, Ratcliffe [35] p. 252 suggests, “involve differences in the types of possibility to which one is receptive”. As such, they are presupposed by and make possible intentional states. They are, in this sense, themselves object-less but make the object-directedness of intentionality possible. You could then think of loneliness as an existential feeling that structures the sufferer’s experience as a whole. In a recent talk, Ratcliffe [36] suggested that many instances of loneliness involve a sense of being unable to belong, of “exclusion from possibilities open to others”. Such a view is well suited to explain both chronic and local experiences of loneliness: it can explain both how the person who is lonely due to the loss of his partner feels excluded from the possibilities offered by the entire life they used to have together, and it can explain also why someone feels a pang of loneliness at a party populated by strangers who all seem to be close friends. The hallmark of this way of understanding loneliness as a mood, and one aspect that distinguishes it from an account modelled on Goldie’s notion of “feeling towards”, is its forward-directedness: it seeks to explain a person’s sense of loneliness not in terms of the experience of her present surroundings, but of future possibilities for engagement with these surroundings.

### 4.3. Relationism and 4-E

A third way of thinking about loneliness is, yet again, inspired by the philosophy of perception. Most contemporary theories of visual perception acknowledge that in the ordinary case, in which things are as they appear to be, the perceiver stands in a relation to the perceived object. Differences arise with regard to the question of how to characterize this relation. In this section, I focus on the so-called “4-E” approach to perception and cognition, according to which someone’s relation to their environment is not best explained by appeal to purely psychological items such as mental representations but involves bodily activity in particular social and physical contexts—it is, variously, “embodied, embedded, enacted, extended” (see, e.g., the contributions in Newen, De Bruin, and Gallagher [37]; for a recent critical review, see Carney [38]). On such a view, visual perception is constituted by the perceiver’s active exploration of the environment; visual objects are presented as offering sensorimotor affordances for action (e.g., Noe [39]. These affordances are contingent on the physiological and psychological makeup of the perceiver: whether a set of stairs is experienced as climbable depends on the length of the perceiver’s legs as much as it does on the height of the stairs. The direct relation between the perceiver and the perceived object consists in the opportunities for action that are afforded by the object specifically to the perceiver (the classical account of the notion of affordance is due to Gibson [40]; Chemero [41] proposes a relational view that sees as affordances as relations between objects and particular perceivers).

One can build on this view to develop an enactivist account of the emotions [42,43]. One way to construe loneliness along these lines is to stress the bodily and interactive dimension of social relations and see loneliness as resulting from a lack of such interaction ([44] pp. 77–78). That interaction matters for building meaningful social relations is not in doubt [44]. The mere presence of others does not amount to companionship and does not usually help the lonely person: one does not feel any less lonely because one watches a crowd of people having fun or follows one’s favorite influencer on Instagram. Accounts of what is missing in loneliness are at pains to stress the importance of meaningful relationships. In the attempt to spell out what such relationships consist in, two considerations are worth noting. First, they involve active involvement with someone else; and secondly, they have an element of reciprocity built into them. The relationship between the participants is of a second- person kind [45]: each directs their attention and care to the other and knows themselves to be at the heart of the other’s attention in turn. One way to account for the importance of this kind of reciprocal connection is by reference to the notion of intersubjectivity, as it is discussed in developmental psychology (e.g., Hobson [46]; Reddy [47]; Trevarthen [48]). Along those lines, communicative interactions between child and caregiver play a crucial role in humans’ social and cognitive development and remain vital throughout life: without them, the rich and social life we enjoy would simply not be thinkable [49]. One can think also that humans’ conception of self is developed in and supported by communicative exchanges with others: who one takes oneself to be, and how comfortable one is with one’s self-image, much depends on the emotional attitudes of other towards oneself [50]. Loneliness, on the embodied and enacted view I am sketching, is at its root a deficiency in the sufferer’s embeddedness in the web of social intersubjectively constituted relations. The sense of being alone is ultimately due to a perceived lack of meaningful interaction that shapes and reflects the person’s view of herself.

This kind of account has two aspects: it focuses both on the role of the other person in creating meaningful relationships and on the self-understanding that is afforded by the other’s engagement with oneself. On such a view, loneliness is to be explained by the interplay of social connection and self-understanding. This interplay is taken up in narrative accounts of sense-making. 4-E approaches often combine embodied accounts of the mind with a stress on the importance of such narratives (e.g., Hutto [51]). Though there is, to my knowledge, as yet no 4-E account of loneliness, such a theory may be well-positioned to accommodate the consideration that experiences of loneliness have two directions: they are both about the absence of other persons and about oneself, as the one suffering from that absence. On such a view, the sense of being lonely is always a sense that I am not appropriately connected to others.

## 5. Practical Implications

The first implication of the considerations offered here is that the distinction between objective social isolation and the subjective experience of loneliness is in need of clarification. Social isolation can never be objective in the sense of being independent of the sufferer’s experience of it. All loneliness is, necessarily, experienced subjectively, but there are objective (environmental) and subjective (psychological) factors that may be contributing to the experience (and often someone’s loneliness will be due to an intricate combination of both). The psychological differentiation I have introduced can help with distinguishing between these factors. Begin with intentionalist views that situate the experience of loneliness in the apprehension of something that is external to the psychological state of the individual. I already highlighted the importance of making precise the intentional object of the sufferer’s experience. An additional consideration that is important for remedial work is how the lonely individual herself describes that object: can the sufferer say exactly what she is missing, or does her loneliness manifest itself in a vague but painful general sense of absence of some social connection that she is unable to specify? These questions are not mere philosophical niceties. Help for someone who feels lonely because of some sharply defined event such as the loss of a partner may look very different from support for someone who experiences herself as being deprived from companionship in a more general sense. In the first case the person is desiring something that cannot be retrieved and may benefit from being supported in finding acceptance of that loss; in the second case a change in the person’s social environment might bring relief. 

Intentionalist accounts apply to particular emotions. Consequently, remedial measures that are designed from the intentionalist perspective will focus on particular aspects of the sufferer’s mental life. But, as we saw in the previous section, one can also think of loneliness as a kind of mood. On that approach, one may not treat loneliness as a singular mental state but rather as situated in the interplay between particular experiences and the evaluative perspective in which they arise and that they affect. Ratcliffe [52] discusses how an emotion such as grief can destabilize and unsettle the entire framework within which we experience the environment as meaningful and rationally structured. This meaning is not propositional or linguistic; it is situated on a pre-reflective, affective level. Additionally, as he points out, a common theme in depression is the sufferer’s need to make sense of their predicament, to understand what is happening to them. If you construe loneliness along related lines, the lonely person may benefit from help in working out not just why she feels lonely but also how the absence of others impacts her entire perspective on herself and the world around her. 

The relational approach to loneliness shares some of its outlook with the mood-based account. Both views stress the situatedness and context-dependence of loneliness. On both views, you cannot adequately account for the experience without considering its relation to and impact on the sufferer’s larger psychological, social and physical environment. But the relational view, or at any rate the version I sketched above, places particular emphasis on, first, social interaction and its bodily aspect; and, secondly, the role of narrative in explaining the sufferer’s understanding of herself as being alone. Hutto and Gallagher ([53] p. 165) stress the connection between social interaction and narrative in therapeutic practice:

“A change in narrative self-understanding can modulate our intersubjective behaviors; a change in bodily practices can transform our narrative self-understanding; a change in worldly circumstances, or mood, or instituted practice can equally affect all the other factors that make us who we are.”

Applied to loneliness, this view suggests that improvements in the sufferer’s social surroundings, so that they afford more, or more satisfying, opportunities for interaction, may profitably go hand in hand with measures that help positively shape the lonely person’s self-narrative. Loneliness, on such a picture, is in many cases not simply the consequence of a lack of social connection, nor is it a subjective way of experiencing one’s relation to one’s surroundings. It is, rather, the result of a complex relation between a lack of opportunities for interaction and the narratives that shape one’s self-understanding as being disjointed from one’s environment, and a promising practical approach may build on the holistic character of the sufferer’s experience.

## 6. Conclusions

While much contemporary research aims at devising means to effectively relieve loneliness, its conceptual dimension remains poorly understood. To aid this investigation, I have classified questions arising for a theory of loneliness along their experiential, social, and normative and cognitive dimensions and have drawn on some considerations from the philosophy of mind to draw up three ways of thinking about the structure of the experience of loneliness. It is vital to stress the preliminary nature of this investigation. Since there are very few conceptually well-worked out accounts of loneliness, this paper cannot do more than provide cursory sketches of possible avenues for further work. Thinking more deeply about all three dimensions of loneliness outlined here is vital for a better understanding of this puzzling and intricate condition and for designing corresponding remedial measures.

## Data Availability

No new data were created or analyzed in this study. Data sharing is not applicable to this article.
